# *Satureja khuzistanica* Essential Oil-Loaded Solid Lipid Nanoparticles Modified With Chitosan-Folate: Evaluation of Encapsulation Efficiency, Cytotoxic and Pro-apoptotic Properties

**DOI:** 10.3389/fchem.2022.904973

**Published:** 2022-06-22

**Authors:** Seyedeh Farnoosh Tabatabaeain, Ehsan Karimi, Mehrdad Hashemi

**Affiliations:** ^1^ Department Genetics, Islamic Azad University, Tehran Medical Branch, Tehran, Iran; ^2^ Department of Biology, Mashhad Branch, Islamic Azad University, Mashhad, Iran; ^3^ Farhikhtegan Medical Convergence Science Research Center, Farhikhtegan Hospital Tehran Medical Sciences, Islamic Azad University, Tehran, Iran

**Keywords:** solid lipid nanoparticles, chitosan, folate, anticancer potential, Satureja khuzistanica essential oil

## Abstract

The study aimed to synthesize *Satureja khuzistanica* essential oil-loaded SLN nanoparticles and to modify the surface of nanoparticles with folate-bound chitosan (SEO-SCF-NPs), and finally to investigate the effects of its toxicity and pro-apoptosis. For this purpose, the SEO-SLN nanoparticles were prepared using stearic acid, lecithin, tween 80, and water by high-pressure homogenization method. After characterization by FTIR, SEM, DLS, and ZETA potential methods, its toxicity effect against normal (HFF) and cancer (MCF-7) cells were evaluated by MTT assay. The occurrence of apoptosis in MCF-7 cells was assessed by flow cytometry and molecular analysis. The obtained results revealed the formation of round nanoparticles with a size of 279.40 nm, single dispersed (PDI: 0.3) and stable (ζ–potential: +31.69 mV). SEO-SCF-NPs indicated the effect of selective toxicity against MCF-7 cells (IC_50_: 88 μg/ml). Molecular analysis showed that SEO-SCF-NPs could inhibit cancer cells by activating the internal pathway of apoptosis as well as cell cycle disruption. Our finding suggests that SEO-SCF-NPs is a suitable candidate for preclinical cancer studies.

## 1 Introduction

Cancer is a syndrome involving a series of diseases caused by the rapid formation and proliferation of abnormal cells with the ability to spread beyond their natural boundaries. This ability leads to the spread of abnormal cells to other tissues in the body, where a phenomenon called metastasis occurs, which is a major cause of cancer death (Pérez-Herrero and Fernández-Medarde, 2015). Surgery as a primary treatment to remove solid mass followed by chemotherapy and radiotherapy are among the most effective current treatments for cancer (Oneda et al., 2020). Induction of apoptosis by disrupting the cell cycle and generating free radicals is one of the chemotherapeutic strategies to inhibit cancer (Gupta et al., 2020). However, the side effects of chemotherapeutic drugs, including adverse effects on fast-growing cells such as blood cells, gastrointestinal tract, hair loss, and most importantly drug resistance, have limited the use of this treatment (Oun et al., 2018). Advances in biomedicine based on the molecular knowledge of many diseases and the identification of cellular as well as molecular processes that lead to various diseases have led to the discovery of therapeutic molecules with a natural origin effectively affecting target cells (Bayón-Cordero et al., 2019). However, in some diseases, including cancer, although new drugs are being researched, they have problems challenging their usage. The low solubility of drugs, their lack of penetration into the cell membrane, their instability, etc. reduce the concentration of the drug at the target site, prompting use of high doses of the drug, followed by high toxicity and side effects (Bayón-Cordero et al., 2019).

Identifying natural compounds with the ability to inhibit cancer cells can be a viable alternative to chemotherapy drugs. Essential oils are plant secondary metabolites with a combination of monoterpenoids and sesquiterpenoids, phenylpropanoids, flavonoids, etc., which have different biological effects such as anti-tumor, antioxidant, and antibacterial effects due to their composition (Souto et al., 2020). The chemical structure of essential oils is unstable and decomposes due to chemical as well as enzymatic reactions in exposure to moisture, heat, oxygen, and light, where the products of decomposition can disrupt their biological properties and function (Pérez-Herrero and Fernández-Medarde, 2015). The use of targeted drug delivery systems (DDS) in addition to protecting the essential oil from redox reactions can reduce side effects of drugs by increasing bioavailability and elevating drug concentration at the site (Li et al., 2012). The choice of drug delivery systems depends on several factors, including the nature of the drug used. Solid lipid (SLN) nanoparticles are a useful option for the transport of plant essential oils (fatty nature) due to their high biocompatibility and degradability, low toxicity, and ability to encapsulate hydrophobic compounds (Teimouri and Odoumizadeh, 2021). SLNs have successfully encapsulated different types of phytochemicals such as curcumin and Marrubiin (Nakhlband et al., 2018; Wang et al., 2018). They enhance the stability of drugs by protecting them against chemical degradation and enhance solubility, biocompatibility, as well as bio-accessibility of the loaded-drugs (Stella et al., 2018).

Encapsulation of compounds in nanometer structures due to enhanced intracellular uptake of the drug is one of the effective methods for targeting (Teimouri and Odoumizadeh, 2021). Other methods include modifying the surface of carriers to boost the sensitivity of drug delivery systems to target areas (Ganesan et al., 2018). The use of chitosan coating to promote the transfer of bioactive compounds has led to the production of the first generation of surface-modified SLN. Chitosan due to its cationic property, high mucosal adhesion, high bioavailability, and low toxicity is known as a suitable coating for SLN nanocarriers for improving the transport of bioactive compounds (Luo et al., 2015; Campos et al., 2017; Sandri et al., 2017; Vijayakumar et al., 2017). Binding of folic acid (FA) ligand to the surface of carrier systems can be effective in targeted drug delivery to cancer cells (FA positive receptor). Binding of FA ligand to the surface of nanocarriers is easily possible and these ligands have been widely used to target various cancer cells due to their non-toxic and non-immunogenic nature as well as high affinity (Pan and Feng, 2014; Dhas et al., 2015).

The present study was performed to investigate the anti-cancer effects of *Satureja khuzistanica* essential oil loaded on SLN nanocarriers. In this study, to boost the efficiency of nanocarriers in targeting breast cancer cells, chitosan coating was used on the nanocarrier surface, after which FA ligand was attached to the carrier surface. After characterizing the nanoparticles, their cytotoxic and pro-apoptotic effects were evaluated by flow cytometry and molecular analysis.

## 2 Material and Methods

### 2.1 Materials

MCF-7 (ATCC HTB-22) as breast cancer cell line and fibroblast cell line HFF (ATCC SCRC-1041) as normal cell line were provided from the Pasteur Institute of Iran Cell Bank. Sigma-Aldrich (United States) provided MTT, PBS tablets, and Propodium iodide. Invitrogene Co. supplied culture medium (RPMI and DMEM), trypsin EDTA and penicillin-streptomycin. FBS was purchased from Gibco (United States). Stearic acid, non-ionic surfactant (Tween 80), Lecithin, DMSO, and ethanol were provided from Merck Co. Finally, Ampliqon supplied the SYBR Green.

### 2.2 SEO-SLN-NPs Preparation

The high-pressure homogenization method was applied to loaded the SEO to SLNs (SEO-SLNs). Specifically, 100 µl of SEO was dissolved in the melted 4% v/v stearic acid and 2% v/v lecithin (85°C). Next, the aqueous phase containing 4% v/v tween 80 heated at 85°C was added to the lipid phase and homogenized for 3 min at 80°C. Here, 1 ml of the final solution was centrifuged and the supernatant was used to evaluate the encapsulation of SEO.

### 2.3 Surface Modification of SEO-Loaded SLN- NPs With CS-FA

In the first step, chitosan was dissolved in acetic acid and then FA was attached to it using NHS (N-Hydroxysuccinimide) and EDC (N-(3-Dimethylaminopropyl)-N′-ethylcarbodimide hydrochloride). For this purpose, 2 mg of FA was dissolved in DMSO. 4 mg of EDC was then dissolved in DMSO and added to FA, and finally the NHS was added to the above solution and incubated for 1 h in the dark. Next, the NHS-FA was filtered and CS was added drop wise and the final pH was set at 9. In order to synthesize SLN, stearic acid and lecithin were transferred to Falcon in a ratio of 2: 1 and incubated at 80°C. Then 100 μL of SEO was added to Falcon. To prepare the aqueous phase, 10 ml of TW 80 was transferred to a falcon and incubated at 80°C, and finally the aqueous phase was added to the lipid phase and homogenized for 3 min. In order to modify the SLN surface, CF solution was gently added to the SLN and after 2 h of incubation on the stirrer, it was centrifuged (18,928 x g). Cell sediment was lyophilized and supernatant was collected to evaluate the encapsulation rate of essential oil and FA binding.

### 2.4 Synthesis Confirmation of SEO-SCF-NPs by DLS, ζ–Potential, FTIR, and SEM

The physicochemical properties such as particle size, PDI, and surface charge of the SEO-SCF-NPs were determined as triplicate at 25 ± 0.5 and 175°C scattering angle using Malvern zeta sizer (Malvern Instruments, United Kingdom) based on the DLS way. SEM imaging method was used to investigate the size and shape of SEO-SCF-NPs. For this purpose, the dispersed nanoparticles were placed on the aluminum page. Once dried, they were coated with gold and examined microscopically. The FTIR method was used to investigate and identify the functional groups in SEO-SCF-NPs. First, 2 mg of SEO-SCF-NPs was mixed with KBr powder and compressed into tablets. The tablet was then analyzed in the FTIR apparatus within 500 cm^−1^ to 4,000 cm^−1^.

### 2.5 SEO Entrapment Efficiency

The amount of encapsulated essential oil in nanoparticles was evaluated indirectly. For this purpose, standard curves were drawn for different concentrations of essential oils (32.5, 62.5, 125, 250 and 500 μg/ml). By substituting the extent of supernatant adsorption (section 2.2) in the formulation obtained from the standard curve, the amount of essential oil in the supernatant was obtained. The following formula was used to calculate the amount of encapsulated essential oil. EE % = entrapped SEO/total SEO×100%.

### 2.6 FA Bonding

The binding of FA to the surface of SEO-SCF-NPs was evaluated using the HPLC method. For this purpose, the supernatant of section 2.3 was used. The level below the peak of the supernatant was evaluated compared to the level below the peak of the standard FA sample. Next, the amount of FA binding was evaluated by considering the volume of the supernatant and the amount of initial FA.

### 2.7 MTT Assay

MTT method was used to evaluate the toxicity of SEO-SCF-NPs on MCF-7 cells compared to HFF. First, a 5*10^3^ of each cell was cultured in eight columns in three replications and after 24 h of incubation, the cells would attach to the bottom of the plate, and were treated serially with different concentrations of SEO-SCF-NPs. After 48 h, the treatment medium was removed from each well and replaced with 20 μL of MTT solution. After 4 h, the MTT solution was drained from each well using a micropipette and replaced with 100 μL DMSO. Finally, the absorbance of the samples was recorded at 570 nm and the % viability of cells was calculated using the following formula. % cells’ Viability = OD _sample_/OD _control_ × 100. In the present study, tamoxifen was applied as a positive control as an anticancer drug.

### 2.8 Evaluation of Cell Death Type

#### 2.8.1 Flow Cytometry

Initially, a certain number of cells were cultured in a six-well plate and after 24 h, they were treated with the concentrations obtained from the MTT test. After 48 h of incubation, the treatment medium was drained and the cells were washed with 1 ml PBS. After draining the PBS, 500 μL of trypsin was added to each well, and after 2 min of incubation, the cells were transferred from each well to separate microtubes. The samples were then centrifuged and after draining the supernatant, 300 μL of Propidium Iodide (PI) dye containing 0.2% Triton X100 was added to the cell sediment; after 10 min of incubation, it was analyzed by flow cytometry.

#### 2.8.2 Molecular Analysis

To investigate the changes in the expression of apoptosis of the genes involved in SEO-SCF-NPs-treated cells, the cells were first cultured in a 12.5 cm^2^ flask. After 24 h, they were treated with a moderate concentration (90 μg/ml) obtained from the MTT test. After 48 h, the treatment medium was drained and after washing with PBS, the total RNA of the cells was extracted according to the protocol of the manufacturer of the Biotech kit. After examining the amount of RNA with the Nano drop device, it was used to synthesize complementary DNA. The reaction mixture containing complementary DNA along with SYBR Green, distilled water, and specific primer with a final volume of 20 μL was prepared and then analyzed under a specific temperature-time program in Bio-Rad Real Time PCR.

### 2.9 Statistical Analysis

The data were analyzed using SPSS (version 21) where *p* < 0.05 was considered significant. The MTT and molecular assay results were analyzed with the One-way ANOVA, LSD test.

## 3 Results

### 3.1 Analysis DLS and ζ–the Potential of SEO-SCF-NPs

**FIGURE 1:**
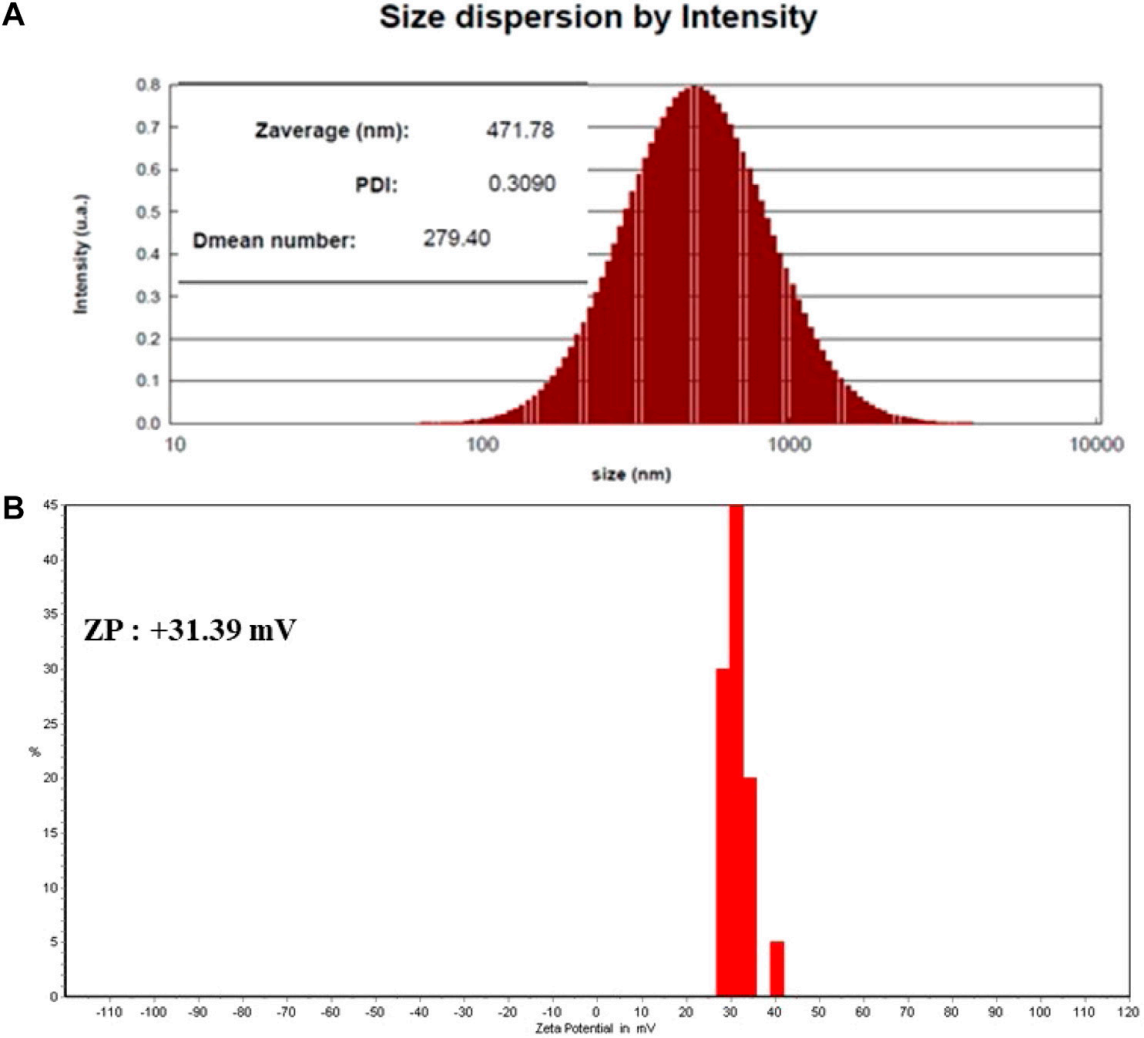
**(A)** DLS analysis (Ps: 279.40 nm and PDI: 0.3) and **(B)** surface charge (+31.69 mV) of SEO-SCF-NPs.

DLS and surface potential profiles of SEO-SCF-NPs are presented in Figure 1. Their particle sizes and PDI were 279.4 nm and 0.3, while the zeta potentials of SEO-SCF-NPs were +31.69 mV. In a nanostructure, particles with the surface charge of 30 mV and PDI <0.7 are considered as stable and homogenous formulations (Kelidari et al., 2021) thus, SEO-SCF-NPs nanoformulation had satisfactory properties.

### 3.2 SEM Electron Microscope Analysis of SEO-SCF-NPs

**FIGURE 2:**
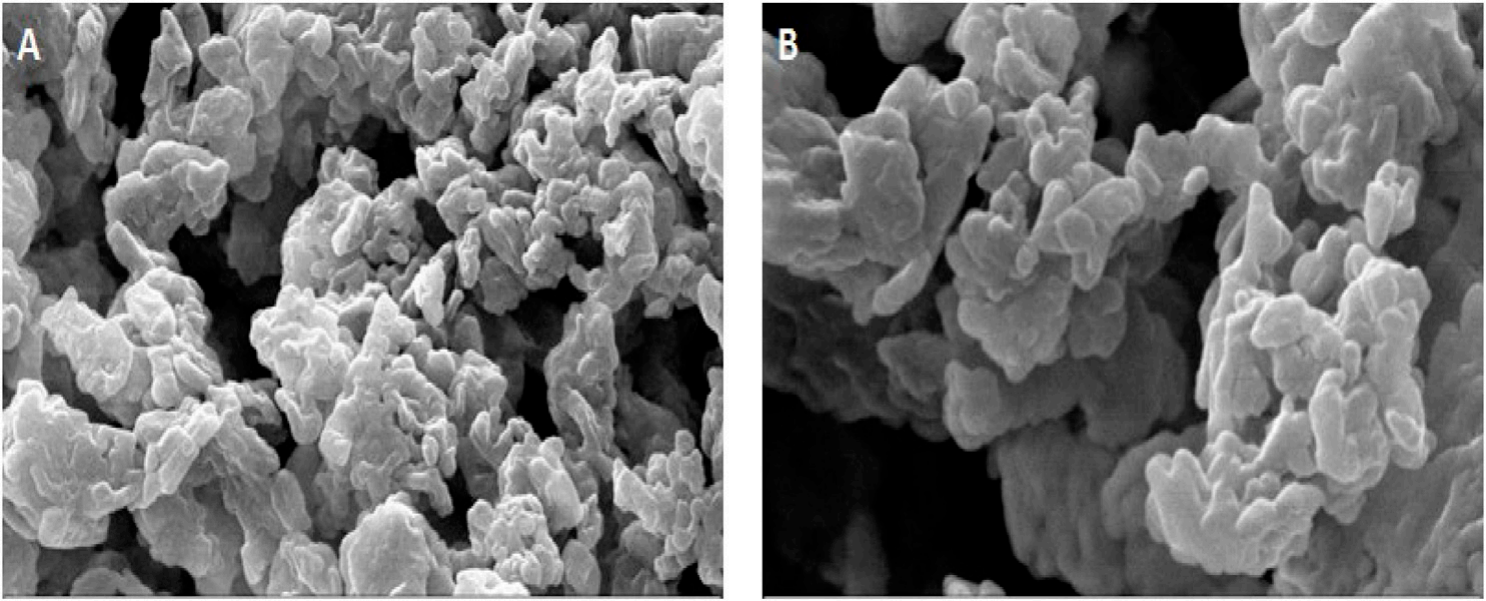
SEM electron microscope image of SEO-SCF-NPs.

The morphology study results of SEO-SCF-NPs with SEM microscope are shown in Figure 2. The images exhibited the spherical shape of the nanoparticles. The particle size shown in the SEM images confirms the DLS results.

### 3.3 FTIR Analysis of SEO-SCF-NPs

**FIGURE 3:**
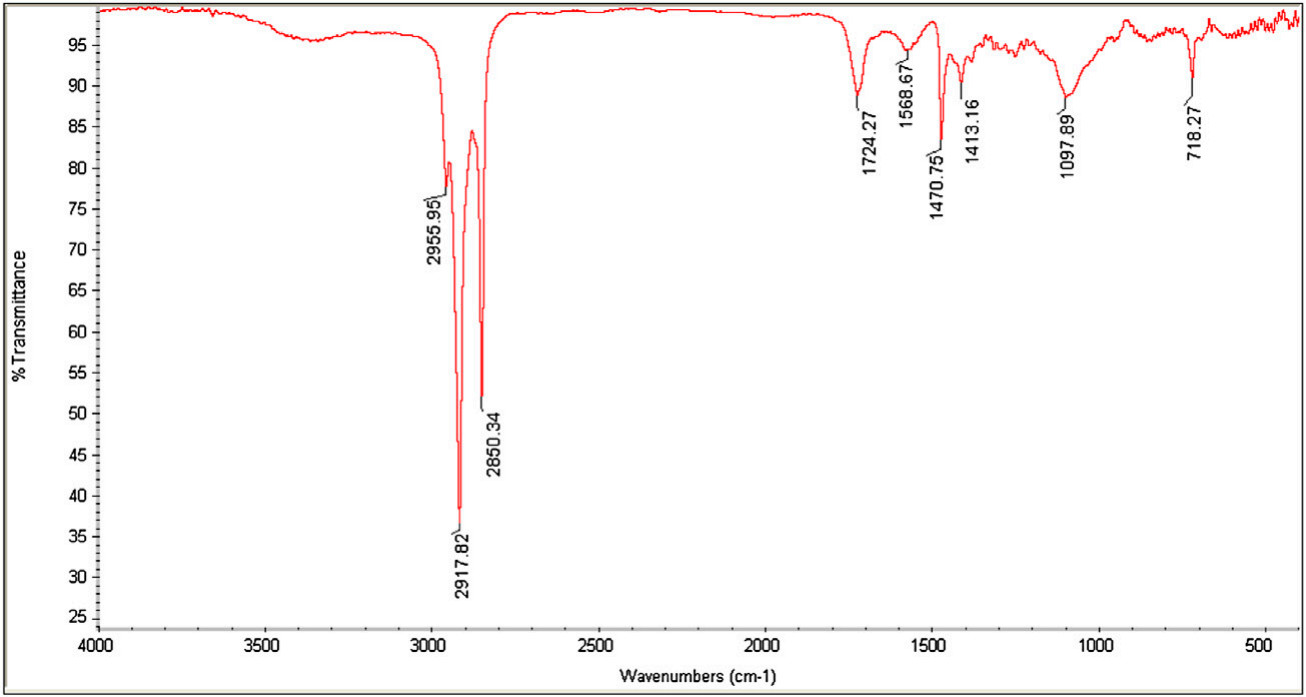
FTIR spectra of SEO-SCF-NPs.

The FTIR spectra for SEO-SCF-NPs are presented in Figure 3. Specific SLN peaks include strong peaks at 2846.9 and 2918 6 cm^−1^ (O-H and C-H bonds), at 1470 and 1728.1 cm^−1^ (C=O and C=C bonds), and 1079 cm^−1^ (C-C and C-O) (Abbasalipo et al., 2016). The formation of FA-CS is indicated by the presence of a peak at 1731 cm^−1^ (C = O), which confirms that CS has successfully modified SLN-NPs (Dhas et al., 2015; Lu et al., 2019). The shift of SLN specific peaks of 2846.9 cm^–1^ and 2918.6 cm^–1^ to 2850.10 cm^–1^ and 2917.886 cm^–1^ plus peak at 1728.1 cm^–1^ to 1724.27 cm^–1^ approved the SLN surface modification and SEO loading.

### 3.4 SEO Entrapment

The standard curve for different concentrations of SEO at 270 nm was obtained with a linear relationship (Y = 0.008x + 0.0075) and a correlation coefficient of 0.921 (R^2^ = 0.92). According to the density of SEO (1000 mg/ml) and incoporating the obtained numbers in the standard curve formula, the value of free SEO was calculated. Through the formula mentioned in section 2.5, SEO encapsulation efficiency was reported to be 84.5%.

### 3.5 FA Bonding

In the HPLC method, by calculating the level of FA in the supernatant and standard FA, the amount of supernatant FA was calculated to be 0.02 mg/ml. Given the volume of supernatant (25 ml) and the amount of primary FA (2 mg), the FA binding percentage was reported 75%.

### 3.6 MTT Assay for SEO-SCF-NPs

**FIGURE 4:**
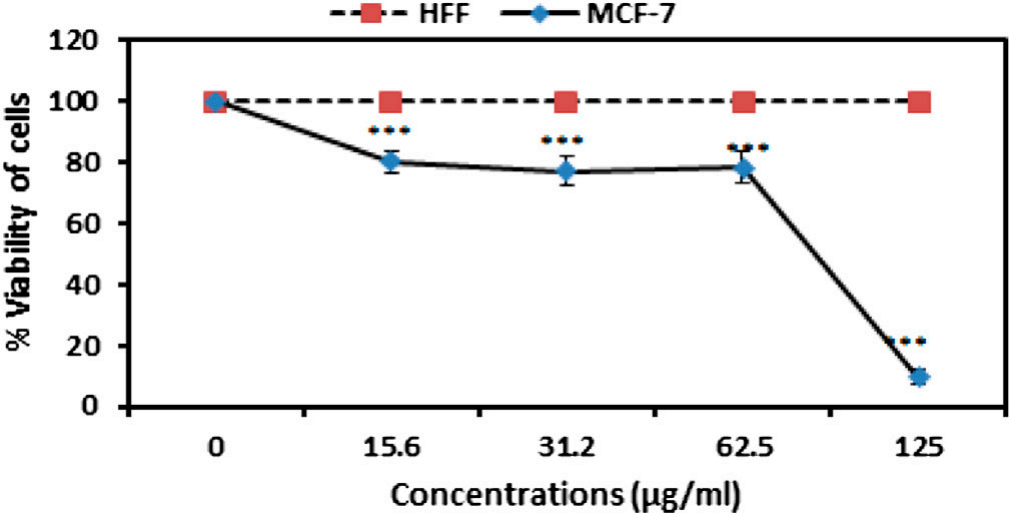
Analysis of MCF-7 and HFF cell viability in treatment with various concentrations of SEO-SCF-NPs. The results were represented as mean ± SD and “***” *p* < 0.001.

Examination of viability alteration of MCF-7 and HFF cell in SEO-SCF-NPs treatment (Figure 4) shows that at all concentrations, the bioavailability of HFF cells was 100% and SEO-SCF-NPs treatment had no inhibitory effect on normal cells. While in MCF-7 cells from the concentration of 15–62 μg/ml, almost 30% of the cells have been inhibited; with increasing the concentration to 125 μg/ml, the inhibition rate has reached above 90%. These results demonstrate the concentration-dependent inhibitory effects of SEO-SCF-NPs against MCF-7 cells. The IC_50_ of SEO-SCF-NPs and tamoxifen as a positive against MCF-7 cells was obtained 88.37 and 34.61 μg/ml respectively.

### 3.7 Flow Cytometry

**FIGURE 5:**
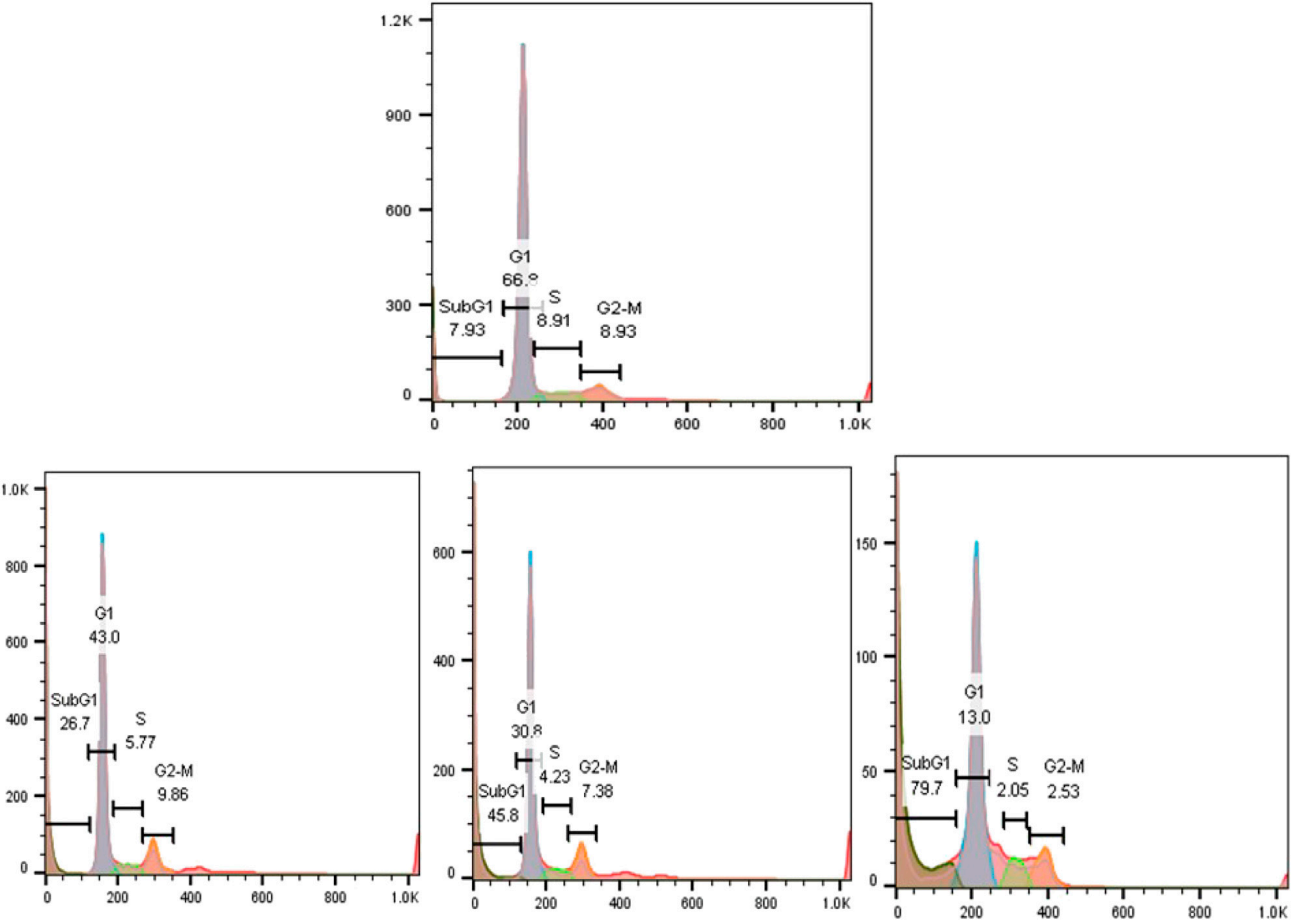
Cell cycle analysis in MCF7 cells treated with different concentrations of SEO-SCF-NPs.

Cell cycle changes in SEO-SCF-NPs-treated cells compared to untreated cells are shown in Figure 5. In the study of the cell cycle, the percentage of Sub-G1 phase cells indicates apoptotic cells. Examination of the cell cycle in untreated cells shows that about 8% of the cells are in this phase, while in the cells treated with nanoparticles with elevation of concentration, the percentage of Sub-G1 phase cells Increased to 26.7, 45.8, and 79.8%. These results confirm the occurrences of apoptosis in nanoparticle-treated cells. No cessation and inhibition of cell growth was observed in any of the other stages of the cell cycle (G1, S, and G2-M). The results generally show that nanoparticles stop cell growth and proliferation by stopping cells in the Sub-G1 phase and causing cell death.

### 3.8 Gene Expression

**FIGURE 6:**
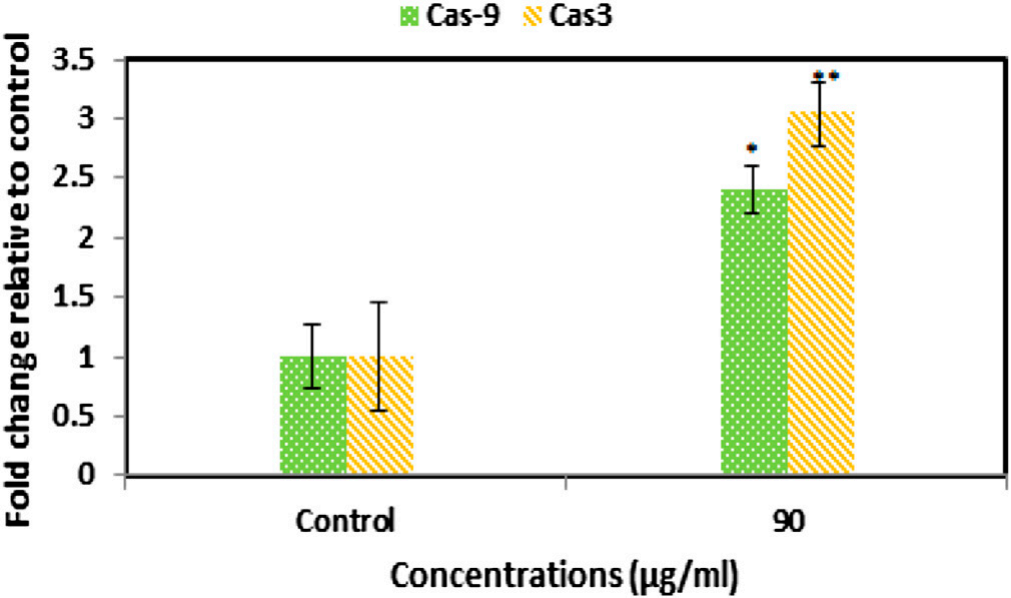
Increased expression of caspase 3 and 9 genes in SEO-SCF-NPs-treated cells by the qPCR method. The results were represented as mean ± SD and “*” *p* < 0.05 and “**” *p* < 0.01.

Expression of caspase genes as the genes involved in the apoptosis pathway in nanoparticle-treated cells showed a significant increase compared to controls (Figure 6). High expression of caspase-9 indicates the occurrence of apoptosis via an intrinsic pathway, which ultimately leads to increased expression of the caspase-3 gene. The results of the molecular analysis confirm the activation of the intrinsic apoptosis pathway.

## 4 Discussion

Solid lipid nanoparticles (SLNs) are lipid-based carriers on a scale of 30–1000 nm which are synthesized using degradable lipids and are therefore safe biological systems. These carriers are one of the most suitable delivery systems for plant and bioactive compounds. The advantages of these nanocarriers include escape from spleen or liver filtration, high bioavailability, less chronic or acute toxicity, as well as the possibility of loading hydrophilic and hydrophobic compounds (Ganesan et al., 2018). Several studies demonstrated that SLN nanocarriers have been used for oral delivery of bioactive plant compounds such as curcumin, quercetin, resveratrol, and especially essential oils. These natural phytochemicals have been widely used all over the world and their use is constantly enhancing due of the strong demand for pure natural ingredients in food and pharmaceutical industries (Ayan et al., 2017; Comoglu et al., 2017; Couto et al., 2017). Burst release of plant bioactive compounds encapsulated in SLN nanoparticles in systemic conditions and internal environment of the body with different pH is one of the disadvantages of using these carriers (Ramalingam et al., 2016). Modification of nanoparticle surface can be effective to enhance the transfer efficiency of bioactive compounds and prevent their explosive release under systemic conditions (Ramalingam and Ko, 2015). In some studies, various compounds such as heparin, polyethylene glycol, albumin, and polysaccharides have been used to modify the surface of SLN nanoparticles to control the transport of bioactive plant compounds (Lee et al., 2014; Niu et al., 2016; Sandri et al., 2017). In the present study, SLN nanocarriers were used to load *Satureja khuzistanica* essential oil. After the synthesis, the surface of nanoparticles was modified using a chitosan coating attached to folic acid. The synthesized nanoparticles had an average diameter of 279.40 nm, a dispersion index of 0.3, and a surface charge of +31.69 mV. Considering the acceptable size (10–300 nm) of nanoparticles for clinical applications (Mattheolabakis et al., 2012; Kobayashi et al., 2014) as well as the standard for dispersion index for (PDI<0.7) mono disperse particles (Irani et al., 2021) along with surface charge higher than positive 30 for stable particles (Ho et al., 2015), it can be stated that the synthesized nanoparticles are monodisperse, stable with an ideal size for therapeutic studies.

Improvement of the transport and stability of encapsulated bioactive compounds in SLN nanocarriers is directly related to the compounds that are used in the formulation such as surfactants, co-surfactants, and lipids as well as their synthesis methods. In this study, similar to some previous studies, stearic acid, lecithin, tween 80, and distilled water (Singh et al., 2014; Andrade et al., 2019; Trucillo and Campardelli, 2019) were used to synthesize SEO-SLN-NPs by the hot homogenization and probe-sonication method (Wang et al., 2016; Sadat Khadem et al., 2021).

The lipid nature of essential oils is the main reason for using the SLN nanocarrier as a lipid-based transport system as it has high efficiency in encapsulating lipophilic compounds such as essential oils (Ganesan et al., 2018). Similar experiments revealed that the application of SLN carriers to transfer different EOs has been reported, which is comparable to the present study. For example, in a study, a combination of frankincense and myrrh essential oils was used to load SLN nanoparticles (FMO-SLNs), where nanoparticles with dimensions of 113 nm and a surface charge of -16 mV with a loading capacity of 80.60% were synthesized (Shi et al., 2012). Comparison of this study with the present study shows that CS-FA coating in the present study is probably the reason for the enlarged SEO-SCF-NPs size. On the other hand, in the present study, the surface charge of SEO-SCF-NPs +30 mV was reported, which indicates the higher stability of SEO-SCF-NPs. On the other hand, the change of negative charge of SLN nanoparticles to positive is due to the presence of CS coating around SEO-SCF-NPs. Comparison of encapsulation efficiency of essential oil shows that in SEO-SCF-NPs, the loading rate is higher than FMO-SLNs, which is probably due to surface changes of SEO-SCF-NPs. In another study in 2021, SLN-NPs containing *Foeniculum vulgar*e essential oil with a size of 55.43 nm and a surface charge of −29.54 mV were synthesized (Sharifalhoseini et al., 2021), which is smaller than SEO-SCF-NPs and has a negative surface charge. The differences can be attributed to the coating of SEO-SCF-NPs with CS-FA. The presence of a positive charge due to chitosan coating on the surface of nanoparticles can play an effective role in the transfer of nanocarriers to negatively charged cancer cells (Wang et al., 2013). The results of a study conducted in 2016 similar to the present study indicated that modifying the surface of SLN nanoparticles with chitosan could enhance the size and change the surface charge from negative to positive. This study also showed that modifying the surface of nanoparticles can significantly reduce drug release and prevent burst drug release (Ying et al., 2011). In another study, SLN-NPs containing cisplatin were modified by CS coating. Comparison of the physicochemical properties of these nanoparticles (PS: 190 nm and Zp: + 22 mV) with SEO-SCF-NPs showed smaller size and surface charge. The increase in the size of SEO-SCF-NPs can be attributed to the presence of FA on the surface of the SEO-SCF-NPs (Wang et al., 2016). In a study, FA alone was used to modify the surface of SLN nanoparticles. The results of this study have also reported an increase in the therapeutic performance of the modified nanoparticles and a change in the size plus surface charge of the nanoparticles after surface modification (Rajpoot and Jain, 2018). The presence of FA on the surface of nanoparticles increases their intracellular uptake by cancer cells with high FA receptor expression. Since MCF-7 has a high expression of FA receptor (Marshalek et al., 2016; Monteiro et al., 2020), in this study, the MCF-7 cell line was used for anti-cancer studies of SEO-SCF-NPs. The results of the MTT test revealed that the SEO-SCF-NPs had a selective effect on the inhibition of MCF-7 cancer cells (IC_50_: 88.37 μg/ml), while no inhibitory effect was observed on HFF cells. In a 2015 study, chitosan-coated SLN nanoparticles were synthesized for cisplatin loading. The results of this study showed an increase in the toxicity of nanoparticles in chitosan coatings compared to uncoated nanoparticles on cervical cells with a median concentration of 0.16 μg/ml (Wang et al., 2016). In addition, in another study, folic acid was used to modify the surface of oxaliplatin-loaded SLN nanoparticles. The results of this study, similar to the previous study, showed enhanced cellular uptake and cytotoxic effects (IC_50_ < 10 μg/ml) compared to unmodified nanoparticles (Rajpoot and Jain, 2018). The results of these studies show that modifying the surface of nanoparticles with chitosan and folic acid alone can have positive effects on increasing the toxicity and intracellular uptake of the drug in cancer cells. In this study, the effects of pro-apoptotic SEO-SCF-NPs on MCF-7 cells were confirmed by their effect on the cell cycle and altered expression of caspase genes. The results of this study showed that SEO-SCF-NPs by stopping cells in the Sub-G1 stage as well as increasing the expression of the *caspase 9* gene as a gene involved in the intrinsic pathway of apoptosis and increasing the expression of the *caspase* 3 gene have led to apoptosis in treated cells. These funding is an agreement with the study have been carried out by Hajipour et al. that indicated the SLN-loaded Ellagic acid (EA) exhibited anticancer properties against the cancer cells by increasing Bax and decreasing BCL2 expression gene. Similarly, a 2012 study showed that SLN nanoparticles loaded with transferrin-conjugated curcumin could inhibit cancer cells by stopping the cell cycle and increasing the expression of caspase genes (Mulik et al., 2012), which is similar to the present study. In another study, the pro-apoptotic effects of Ellagic acid acid-loaded SLN nanoparticles were confirmed by increasing *Bax* gene expression and reducing the Bcl-2 gene expression (Hajipour et al., 2018). In contrast to the present study, a 2019 study showed that tamoxifen-loaded SLN nanoparticles could inhibit breast cancer cells in a concentration- and time-dependent manner without stopping the cell cycle (Abbasalipourkabir et al., 2016). Liang et al. reported the pro-apoptotic effect of SLN nanoparticles via activating the internal apoptotic pathway and disrupting the cell cycle. In this study, it was shown that treatment with nanoparticles in macrophage cells caused an imbalance in Bax/Bcl-2 expression as well as increased Caspase3 plus PARP expression (Liang et al., 2019). In a study in 2021, the effect of SLN-NPs loaded with *Foeniculum vulgare* essential oil on MCF-7 cancer cells by stopping cells in the Sub-G1 phase was confirmed by flow cytometry (Sharifalhoseini et al., 2021), which is similar to the present study.

## 5 Conclusion

The results of this study confirmed the formation of a stable (+31.69 mV) and single-dispersed (PDI: 0.3) SEO-SCF-NPs on the nanometer scale (279.40 nm). In examining the toxicity effect of SEO-SCF-NPs, the selective toxicity of nanoparticles against breast cancer cells compared to normal cells has been reported. Further investigation showed that one of the inhibitory mechanisms of nanoparticles on cancer cells is through activation of the internal pathway of apoptosis and cell cycle disruption. These results suggest the use of SEO-SCF-NPs as a suitable candidate for preclinical cancer studies.

## Data Availability

The original contributions presented in the study are included in the article/supplementary material, further inquiries can be directed to the corresponding author.

## References

[B1] Abbasalipo R. Fallah M. Sedighi F. Maghsood A. H. Javid S. (2016). Nanocapsulation of Nitazoxanide in Solid Lipid Nanoparticles as a New Drug Delivery System and *In Vitro* Release Study. J. Biol. Sci. 16 (4), 120–127. 10.3923/jbs.2016.120.127

[B2] Abbasalipourkabir R. Salehzadeh A. Abdullah R. (2016). Tamoxifen-loaded Solid Lipid Nanoparticles-Induced Apoptosis in Breast Cancer Cell Lines. J. Exp. Nanosci. 11 (3), 161–174. 10.1080/17458080.2015.1038660

[B3] Andrade L. N. Oliveira fnm. Chaud fnm. Alves fnm. Nery fnm. da Silva fnm. (2019). Praziquantel-solid Lipid Nanoparticles Produced by Supercritical Carbon Dioxide Extraction: Physicochemical Characterization, Release Profile, and Cytotoxicity. Molecules 24 (21), 3881. 10.3390/molecules24213881 PMC6864877 31661906

[B4] Ayan A. K. Yenilmez A. Eroglu H. (2017). Evaluation of Radiolabeled Curcumin-Loaded Solid Lipid Nanoparticles Usage as an Imaging Agent in Liver-Spleen Scintigraphy. Mater. Sci. Eng. C 75, 663–670. 10.1016/j.msec.2017.02.114 28415513

[B5] Bayón-Cordero L. Alkorta I. Arana L. (2019). Application of Solid Lipid Nanoparticles to Improve the Efficiency of Anticancer Drugs. Nanomaterials 9 (3), 474. 10.3390/nano9030474 PMC6474076 30909401

[B6] Campos J. Varas-Godoy M. Haidar Z. S. (2017). Physicochemical Characterization of Chitosan-Hyaluronan-Coated Solid Lipid Nanoparticles for the Targeted Delivery of Paclitaxel: a Proof-Of-Concept Study in Breast Cancer Cells. Nanomedicine 12 (5), 473–490. 10.2217/nnm-2016-0371 28181464

[B7] Comoglu T. Arisoy S. Akkus Z. (2017). Nanocarriers for Effective Brain Drug Delivery. Curr. Top. Med. Chem. 17 (13), 1490–1506. 10.2174/1568026616666161222101355 28017157

[B8] Couto R. Alvarez V. Temelli F. (2017). Encapsulation of Vitamin B2 in Solid Lipid Nanoparticles Using Supercritical CO 2. J. Supercrit. Fluids 120, 432–442. 10.1016/j.supflu.2016.05.036

[B9] Dhas N. L. Ige P. P. Kudarha R. R. (2015). Design, Optimization and *In-Vitro* Study of Folic Acid Conjugated-Chitosan Functionalized PLGA Nanoparticle for Delivery of Bicalutamide in Prostate Cancer. Powder Technol. 283, 234–245. 10.1016/j.powtec.2015.04.053

[B11] Ganesan P. Ramalingam P. Karthivashan G. Ko Y. T. Choi D.-K. (2018). Recent Developments in Solid Lipid Nanoparticle and Surface-Modified Solid Lipid Nanoparticle Delivery Systems for Oral Delivery of Phyto-Bioactive Compounds in Various Chronic Diseases. Int. J. Nanomedicine 13, 1569–1583. 10.2147/ijn.s155593 29588585 PMC5858819

[B12] Gupta N. Verma K. Nalla S. Kulshreshtha A. Lall R. Prasad S. (2020). Free Radicals as a Double-Edged Sword: The Cancer Preventive and Therapeutic Roles of Curcumin. Molecules 25 (5), 5390. 10.3390/molecules25225390 PMC7698794 33217990

[B13] Hajipour H. Hamishehkar H. Rahmati-yamchi M. Shanehbandi D. Nazari Soltan Ahmad S. Hasani A. (2018). The Enhanced Anti-cancer Capability of Ellagic Acid Using Solid Lipid Nanoparticles (SLNs). Int. J. Cancer Manag. 11 (1), 9402. 10.5812/ijcm.9402

[B14] Ho H. N. Tran T. H. Tran T. B. Yong C. S. Nguyen C. N. (2015). Optimization, and Characterization of Artesunate-Loaded Chitosan-Decorated Poly (D, L-Lactide-Co-Glycolide) Acid Nanoparticles. J. Nanomater. 2015, 1–12. 10.1155/2015/674175

[B15] Irani M. Tabrizi M. H. Ardalan T. Nosrat T. (2021). Artemisia Vulgaris Essential Oil Nanoemulsions (AVEO-NE), a Novel Anti-angiogenic Agent and Safe Apoptosis Inducer in MCF-7 Human Cancer Cells. Inorg. Nano-Metal Chem. 52, 1–12. 10.1080/24701556.2021.1980022

[B16] Kelidari H. R. Alipanah H. Roozitalab G. Ebrahimi M. Osanloo M. (2021). Anticancer Effect of Solid-Lipid Nanoparticles Containing Mentha Longifolia and Mentha Pulegium Essential Oils: *In Vitro* Study on Human Melanoma and Breast Cancer Cell Lines. Bioint. Res. App. Chem. 12, 2128–2137. 10.33263/briac122.21282137

[B17] Kobayashi H. Watanabe R. Choyke P. L. (2014). Improving Conventional Enhanced Permeability and Retention (EPR) Effects; what Is the Appropriate Target? Theranostics 4 (1), 81–89. 10.7150/thno.7193 PMC3881228 24396516

[B18] Lee W.-H. Loo C.-Y. Young P. M. Traini D. Mason R. S. Rohanizadeh R. (2014). Recent Advances in Curcumin Nanoformulation for Cancer Therapy. Expert Opin. drug Deliv. 11 (8), 1183–1201. 10.1517/17425247.2014.916686 24857605

[B19] Li K.-K. Yin S.-W. Yang X.-Q. Tang C.-H. Wei Z.-H. (2012). Fabrication and Characterization of Novel Antimicrobial Films Derived from Thymol-Loaded Zein-Sodium Caseinate (SC) Nanoparticles. J. Agric. Food Chem. 60 (46), 11592–11600. 10.1021/jf302752v 23121318

[B20] Liang W.-L. Xiao L. Gu H.-W. Li X.-J. Li Y.-S. Zhang W. K. (2019). Solid Lipid Nanoparticle Induced Apoptosis of Macrophages via a Mitochondrial-dependent Pathway *In Vitro* and *In Vivo* . Int. J. Nanomedicine 14, 3283–3295. 10.2147/ijn.s200395 31123400 PMC6511261

[B21] Lu B. Lv X. Le Y. (2019). Chitosan-modified PLGA Nanoparticles for Control-Released Drug Delivery. Polymers 11 (2), 304. 10.3390/polym11020304 PMC6419218 30960288

[B22] Luo Y. Teng Z. Li Y. Wang Q. (2015). Solid Lipid Nanoparticles for Oral Drug Delivery: Chitosan Coating Improves Stability, Controlled Delivery, Mucoadhesion and Cellular Uptake. Carbohydr. Polym. 122, 221–229. 10.1016/j.carbpol.2014.12.084 25817662

[B23] Marshalek J. P. Sheeran P. S. Ingram P. Dayton P. A. Witte R. S. Matsunaga T. O. (2016). Intracellular Delivery and Ultrasonic Activation of Folate Receptor-Targeted Phase-Change Contrast Agents in Breast Cancer Cells *In Vitro* . J. Control. Release 243, 69–77. 10.1016/j.jconrel.2016.09.010 27686582 PMC5191940

[B24] Mattheolabakis G. Rigas B. Constantinides P. P. (2012). Nanodelivery Strategies in Cancer Chemotherapy: Biological Rationale and Pharmaceutical Perspectives. Nanomedicine 7 (10), 1577–1590. 10.2217/nnm.12.128 23148540

[B25] Monteiro C. A. P. Oliveira A. D. P. R. Silva R. C. Lima R. R. M. Souto F. O. Baratti M. O. (2020). Evaluating Internalization and Recycling of Folate Receptors in Breast Cancer Cells Using Quantum Dots. J. Photochem. Photobiol. B Biol. 209, 111918. 10.1016/j.jphotobiol.2020.111918 32531690

[B26] Mulik R. S. Mönkkönen J. Juvonen R. O. Mahadik K. R. Paradkar A. R. (2012). Apoptosis-induced Anticancer Effect of Transferrin-Conjugated Solid Lipid Nanoparticles of Curcumin. Cancer Nanotechnol. 3 (1), 65–81. 10.1007/s12645-012-0031-2 26069496 PMC4452039

[B27] Nakhlband A. Eskandani M. Saeedi N. Ghafari S. Omidi Y. Barar J. (2018). Marrubiin-loaded Solid Lipid Nanoparticles' Impact on TNF-α Treated Umbilical Vein Endothelial Cells: A Study for Cardioprotective Effect. Colloids Surfaces B Biointerfaces 164, 299–307. 10.1016/j.colsurfb.2018.01.046 29413609

[B28] Niu Z. Conejos-Sánchez I. Griffin B. T. O’Driscoll C. M. Alonso M. J. (2016). Lipid-based Nanocarriers for Oral Peptide Delivery. Adv. drug Deliv. Rev. 106, 337–354. 10.1016/j.addr.2016.04.001 27080735

[B29] Oneda E. Abu Hilal M. Zaniboni A. (2020). Biliary Tract Cancer: Current Medical Treatment Strategies. Cancers 12 (5), 1237. 10.3390/cancers12051237 PMC7281170 32423017

[B30] Oun R. Moussa Y. E. Wheate N. J. (2018). The Side Effects of Platinum-Based Chemotherapy Drugs: a Review for Chemists. Dalton Trans. 47, 6645–6653. 10.1039/c8dt00838h 29632935

[B10] Pan J. Feng S.-S. (2014). “Targeting and Imaging Cancer Cells by Folate-Decorated, Quantum Dots–Loaded Nanoparticles of Biodegradable Polymers,” in Chemotherapeutic Engineering (Jenny Stanford Publishing), 588–607.

[B31] Pérez-Herrero E. Fernández-Medarde A. (2015). *Advanced Targeted Therapies in Cancer: Drug Nanocarriers, the Future of* Chemotherapy. Eur. J. Pharm. Biopharm. 93, 53–79. 10.1016/j.ejpb.2015.03.018 25813885

[B32] Rajpoot K. Jain S. K. (2018). Colorectal Cancer-Targeted Delivery of Oxaliplatin via Folic Acid-Grafted Solid Lipid Nanoparticles: Preparation, Optimization, and *In Vitro* Evaluation. Artif. cells, nanomedicine, Biotechnol. 46 (6), 1236–1247. 10.1080/21691401.2017.1366338 28849671

[B33] Ramalingam P. Ko Y. T. (2015). Enhanced Oral Delivery of Curcumin from N-Trimethyl Chitosan Surface-Modified Solid Lipid Nanoparticles: Pharmacokinetic and Brain Distribution Evaluations. Pharm. Res. 32 (2), 389–402. 10.1007/s11095-014-1469-1 25082210

[B34] Ramalingam P. Yoo S. W. Ko Y. T. (2016). Nanodelivery Systems Based on Mucoadhesive Polymer Coated Solid Lipid Nanoparticles to Improve the Oral Intake of Food Curcumin. Food Res. Int. 84, 113–119. 10.1016/j.foodres.2016.03.031

[B35] Sadat Khadem F. Es-Haghi A. Homayouni Tabrizi M. Shabestarian H. (2021). The Loaded Ferula Assa-Foetida Seed Essential Oil in Solid Lipid Nanoparticles (FSEO-SLN) as the Strong Apoptosis Inducer Agents in Human NTERA-2 Embryocarcinoma Cells. Mater. Technol., 1–9. 10.1080/10667857.2021.1924436

[B36] Sandri G. Motta S. Bonferoni M. C. Brocca P. Rossi S. Ferrari F. (2017). Chitosan-coupled Solid Lipid Nanoparticles: Tuning Nanostructure and Mucoadhesion. Eur. J. Pharm. Biopharm. 110, 13–18. 10.1016/j.ejpb.2016.10.010 27989765

[B37] Sharifalhoseini M. Es‐haghi A. Vaezi G. Shajiee H. (2021). Biosynthesis and Characterisation of Solid Lipid Nanoparticles and Investigation of Toxicity against Breast Cancer Cell Line. IET Nanobiotechnol. 15 (8), 654–663. 10.1049/nbt2.12062 34694719 PMC8675850

[B38] Shi F. Zhao J. H. Liu Y. Wang Z. Zhang Y. T. Feng N. P. (2012). Preparation and Characterization of Solid Lipid Nanoparticles Loaded with Frankincense and Myrrh Oil. Int. J. Nanomedicine 7, 2033–2043. 10.2147/IJN.S30085 22619540 PMC3356207

[B39] Singh B. Vuddanda P. R. M.R. V. Kumar V. Saxena P. S. Singh S. (2014). Cefuroxime Axetil Loaded Solid Lipid Nanoparticles for Enhanced Activity against *S. aureus* Biofilm. Colloids Surfaces B Biointerfaces 121, 92–98. 10.1016/j.colsurfb.2014.03.046 24945607

[B40] Souto E. B. Severino P. Marques C. Andrade L. N. Durazzo A. Lucarini M. (2020). Croton Argyrophyllus Kunth Essential Oil-Loaded Solid Lipid Nanoparticles: Evaluation of Release Profile, Antioxidant Activity and Cytotoxicity in a Neuroblastoma Cell Line. Sustainability 12 (18), 7697. 10.3390/su12187697

[B41] Stella B. Peira E. Dianzani C. Gallarate M. Battaglia L. Gigliotti C. (2018). Development and Characterization of Solid Lipid Nanoparticles Loaded with a Highly Active Doxorubicin Derivative. Nanomaterials 8 (2), 110. 10.3390/nano8020110 PMC5853741 29462932

[B42] Teimouri M. Odoumizadeh M. (2021). Cytotoxicity of Artemisia Vulgaris Essential Oil Encapsulated in SLN on Breast Cancer Cell Line (MCF7). Archives Adv. Biosci. 12 (3), 11–26. 10.22037/aab.v12i3.34543

[B43] Trucillo P. Campardelli R. (2019). Production of Solid Lipid Nanoparticles with a Supercritical Fluid Assisted Process. J. Supercrit. Fluids 143, 16–23. 10.1016/j.supflu.2018.08.001

[B44] Vijayakumar A. Baskaran R. Jang Y. S. Oh S. H. Yoo B. K. (2017). Quercetin-loaded Solid Lipid Nanoparticle Dispersion with Improved Physicochemical Properties and Cellular Uptake. Aaps Pharmscitech 18 (3), 875–883. 10.1208/s12249-016-0573-4 27368922

[B45] Wang Y. Li P. Kong L. (2013). Chitosan-Modified PLGA Nanoparticles with Versatile Surface for Improved Drug Delivery. Aaps Pharmscitech 14 (2), 585–592. 10.1208/s12249-013-9943-3 23463262 PMC3665987

[B46] Wang J. Y. Wang Y. Meng X. (2016). Chitosan Nanolayered Cisplatin-Loaded Lipid Nanoparticles for Enhanced Anticancer Efficacy in Cervical Cancer. Nanoscale Res. Lett. 11 (1), 524–528. 10.1186/s11671-016-1698-9 27888498 PMC5124019

[B47] Wang W. Chen T. Xu H. Ren B. Cheng X. Qi R. (2018). Curcumin-Loaded Solid Lipid Nanoparticles Enhanced Anticancer Efficiency in Breast Cancer. Molecules 23 (7), 1578. 10.3390/molecules23071578 PMC6099699 29966245

[B48] Ying X.-Y. Cui D. Yu L. Du Y.-Z. (2011). Solid Lipid Nanoparticles Modified with Chitosan Oligosaccharides for the Controlled Release of Doxorubicin. Carbohydr. Polym. 84 (4), 1357–1364. 10.1016/j.carbpol.2011.01.037

